# Development of Organic Sourdough Bread with Paste from Germinated Seeds

**DOI:** 10.3390/foods14183263

**Published:** 2025-09-20

**Authors:** Alberto Akiki, Yasmin Muhammed Refaie Muhammed, Fabio Minervini, Ivana Cavoski

**Affiliations:** 1 CIHEAM-MAIB, Mediterranean Agronomic Institute of Bari, 70010 Valenzano, Italy; alberto.akiki@iusspavia.it (A.A.); cavoski@iamb.it (I.C.); 2 University School for Advanced Studies IUSS Pavia, 27100 Pavia, Italy; 3 Department of Bioscience and Technologies for Food, Agriculture, and Environment, University of Teramo, 64100 Teramo, Italy; 4 Dipartimento di Scienze del Suolo, della Pianta e degli Alimenti, Università degli Studi di Bari Aldo Moro, 70126 Bari, Italy; yasmin.muhammed@uniba.it

**Keywords:** germination, wheat, lentils, fiber, bread-making, lactic acid bacteria, yeasts

## Abstract

This study aimed to (i) investigate the effect of using grape water in the production of traditional sourdough; (ii) select seeds for use in laboratory-scale sourdough bread production; and (iii) assess the effect of incorporating fresh germinated seeds into recipe of organic sourdough bread on nutritional, technological, and sensory properties. The pH of both control (CSD, flour only) and boosted (BSD, supplemented with “grape water”) sourdough fell below 4.5 by day 3. After 10 days of back-slopping and fermentation, both sourdoughs harbored 9 log CFU/g of lactic acid bacteria, whereas yeast cell density in the CSD was 1 log cycle higher. Based on their high germination rates (~90%), lentil and wheat seeds were selected as additional ingredients (5%). Bread with germinated lentils (GL) and bread with germinated wheat (GW) were compared with control bread (without seeds). GL and GW breads showed gas cell areas of 28.6% and 18.1%, respectively, which were higher than the control. In addition, GL and GW received higher scores for taste (8.6) and softness (5.6), respectively. Additionally, GL contained more proteins (9.9%) and fewer lipids (0.3%) than the two other bread types, in addition to being potentially labeled as a “source of fiber”.

## 1. Introduction

The number of people who pay special attention to the overall quality of food, especially staple food items, is continuously on the rise [1]. Consumer demand for local organic food, in particular, is also rising due to its perceived health and sustainability benefits. So, nowadays making use of new strategies such as foods with ‘marketed patrimonies’ for alternative sustainable products is essential [2]. Health-centric attributes are the second most significant factor, after taste, that consumers consider when choosing plant-based food items [3]. Bread is one of the most widespread staple foods [4,5]. Although baker’s yeast is frequently used to obtain bread in a short time [6], sourdough is used as an alternative leavening agent that improves bread quality at different layers: (i) it reinforces dough structure [7]; (ii) enriches aromatic profile [8]; (iii) improves nutritional profile [9]; and (iv) extends shelf-life [10]. Indeed, in Europe, at least 30% of bread production incorporates sourdough as either a leavening agent or a genuine improver [11]. Although different commercial preparations of sourdough are available [12], traditional sourdough (also known as type 1 sourdough) is usually obtained upon an initial “spontaneous” fermentation of flour and water, relying on lactic acid bacteria (LAB) and yeasts contaminating flour and the environment of production. Additional ingredients, such as yogurt, grapes, apples [13], water from macerated fruits, grape must [14], and others may also be used because they enrich the dough ecosystem with pro-technological microorganisms (e.g., yeasts) and readily fermentable nutrients [15]. After initial fermentation, a back-slopping process is applied: a portion of the fermented dough is used to inoculate a fresh batch of flour and water (“refreshment”), which undergoes a second fermentation. Repeating these refreshment-fermentation steps daily, while maintaining constant fermentation conditions, the microbial community evolves primarily through dough acidification (attributed to lactic acid fermentation), resulting in a relatively stable ecosystem known as “mature sourdough,” which is dominated by LAB and yeasts [16]. The use of different additional ingredients could either affect the time to reach the low values of pH that characterize mature sourdough [13,14] or boost the technological performances of sourdough [17].

In the last decade, the incorporation of sprouted grains into cereal-based food products has garnered attention due to their potential to enhance mineral and vitamin bioavailability, while reducing anti-nutritional factors [18]. Numerous studies have shown that the incorporation of flour from germinated seeds (sorghum, quinoa, chickpea, wheat) in sourdough bread enhanced its nutritional content, flavor, and texture [19,20,21,22]. Recently, Bresciani et al. [23] compared germinated chickpea flour and grits (coarsely ground hulled seeds) to evaluate their effect in bread containing 25% of these additional ingredients. They found that, compared to the use of flour from germinated chickpeas, the use of grits led to an increase in dough development time and stability; at the same time, bread enriched with grits had a higher specific volume and softer crumbs than bread enriched with chickpea flour [23]. Incorporation of germinated seeds (not milled to flour) could emerge as a viable strategy for improving the nutritional and functional attributes of sourdough bread. In addition, since consumption of fresh sprouts has been associated with foodborne diseases [24], we hypothesize that incorporating them into bread could fulfill food safety requirements because oven baking could inactivate pathogenic bacteria. Furthermore, the use of germinated seeds could be more convenient than “germinated” flour because in this way, two steps (drying and milling) would be skipped. To our knowledge, no study has so far investigated the effects of incorporating germinated seeds on sourdough bread quality.

This study aimed to (i) investigate the effect of using water that had been in contact with raisins in the production of traditional sourdough; (ii) select seeds for use in laboratory-scale sourdough bread production; and (iii) assess the effect of incorporating fresh germinated seeds into recipe of sourdough bread on nutritional, technological, and sensory properties. All ingredients used in this study were from organic farming. Although previous studies considered the use of additional ingredients in the production of traditional sourdough [13,14,15,17], no study so far has compared the effect from the addition of water from raisins on sourdough with that of tap water.

## 2. Materials and Methods

### 2.1. Materials

Seeds from lentils (*Lens culinaris* ssp. *culinaris*), einkorn wheat (*Triticum monococcum*), barley (*Hordeum vulgare*), and bread wheat (*Triticum aestivum* L., cultivar Gentil rosso) were provided by Perniola Alimenti (Rutigliano, Bari, Italy). Bread wheat (cultivar Maiorca) wholemeal flour was produced by Perniola Alimenti. Dried Corinthian raisins and commercial baker’s yeast were purchased from the local market. All raw materials originated from organic farming. Seeds and dried grapes were vacuum-packaged and stored at 25 °C.

### 2.2. Sourdough Preparation

**Figure 1:**
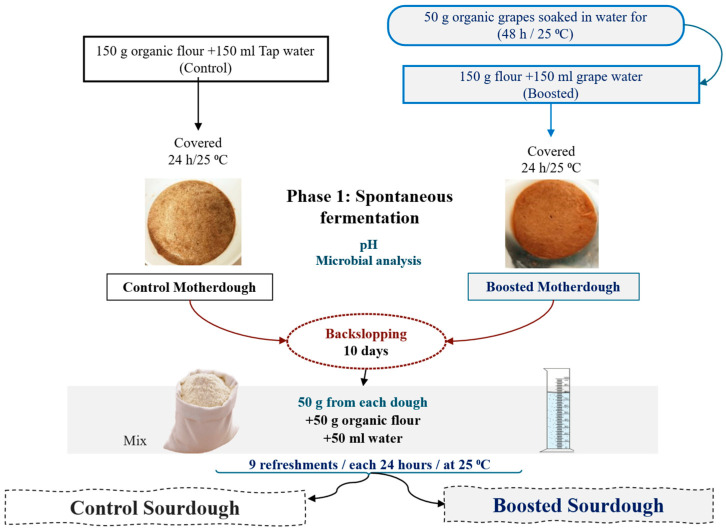
Production of control and boosted sourdough.

Two kinds of type 1 (traditional) sourdough (classified according to De Vuyst et al. [25] were prepared starting from bread wheat flour (150 g): (i) control (CSD), produced by hand-kneading flour with tap water (150 mL); (ii) “boosted” (BSD), produced by hand-kneading flour with 150 mL of “grape water”. Dough Yield was 200. Grape water had been previously obtained upon soaking 50 g of dried grapes in 250 mL of tap water and discarding the grapes after incubation (at room temperature, 48 h). Both doughs were inserted into a plastic jar, covered by a lid, and incubated at 25 °C (relative humidity: 80%) for 24 h. Subsequently, they were back-slopped, inoculating 50 g of the spontaneously fermented dough into a fresh dough, composed of 50 g of flour and 50 mL of tap water (inoculum ratio: about 33%), and then incubated (25 °C, 24 h) (Figure 1).

This back-slopping-and-fermentation step was repeated for at least nine days. BSD corresponded to the protocol usually adopted by Perniola Alimenti. The pH of doughs was measured every day (except for the first spontaneous fermentation) after incubation by directly inserting a Foodtrode electrode (Denver Instrument, Denver, CO, USA). The acidification rate was calculated using the following formula: ΔpH/ΔT, wherein ΔpH represents the difference between the pH value measured after one day and the pH value measured after 10 days of fermentation (pH_1_–pH_10_), and ΔT represents the difference in fermentation times (T10-T1). Microbiological analyses were performed on fermented doughs, as detailed below, after one, three, five, seven, and ten days.

### 2.3. Seed Germination

The germination protocol was adapted from that described by Yang et al. [26], with modifications tailored to optimize sprouting. The seeds were first thoroughly washed under tap water at approximately 15 °C for 20 to 30 min to remove impurities. Afterwards, seeds (25 g) were inserted in a jar with 50 mL of water and soaked separately for 5 h at 25 °C. After soaking, excess water was drained, and the seeds were collected and put on tissue paper. Then, seeds were incubated in a thermostat set at 25 °C in the dark. Relative humidity inside the thermostat during incubation was not controlled; however, seeds were regularly sprinkled with tap water at 12 h intervals to maintain moisture. Sprouting was interrupted when primary roots reached ~5 mm (24 h) to limit exposure to warm, humid conditions that can significantly increase pathogenic bacteria and to align with EFSA guidelines recommending collecting sprouts before the development of leaves [27].

The percentage of germination (% G) was calculated using Equation (1):

% G = germinated seeds × 100/total seeds(1)

### 2.4. Bread-Making

Sourdough bread was made using the BSD and germinated wheat and lentils, according to the recipe provided by Perniola Alimenti, with some modifications. Before bread-making, germinated seeds were transferred into a meat blender machine (Trita Express, RGV S.r.l., Treviglio, BG, Italy) to form a homogenous paste. The blending process commenced by starting at a low speed and gradually increasing the speed. Seed paste was used fresh in bread-making. To prepare one loaf of 500 g, 246.7 g of bread wheat flour (49.34%), 5.05 g of sodium chloride (1.01%), and 25 g (5%) of germinated (lentils or wheat) paste were mixed in a clean and sterilized bowl of a planetary mixer (Orbital stand mixer, model EKM4000, Electrolux AB, Stockholm, Sweden). Subsequently, 74.1 g (14.82%) of BSD, 0.95 g (0.19%) of baker’s yeast, and 148.2 mL of water were added to the bowl, and the mixture was kneaded (5 min) to create dough. In parallel, a control sourdough bread was prepared using 271.7 g of flour and the other ingredients listed above, except for germinated seed paste, which was not added. Each dough was then divided into four loaves and carefully placed onto baking trays lined with parchment paper. Following this, the trays were allowed to undergo a 2 h fermentation at 38 °C (relative humidity: 80%). The fermented loaves were baked in a preheated (to 220 °C) oven for 20 min. Upon completion of the baking process, the bread samples were taken out of the oven and cooled at room temperature for 3 h. In total, twelve loaves were prepared, corresponding to four loaves of each type: control, germinated lentils (GL), and germinated wheat (GW) bread.

### 2.5. Microbiological Analyses

Viable populations of LAB and yeasts were estimated on tap water, grape water, and dough/sourdough, using plate count with selective culture media. Before inoculating plates, 10 g of dough/sourdough samples were homogenized with 90 mL of sterile saline solution (0.9% NaCl) using a stomacher for 3 min. Homogenized samples, as well as tap water and grape water, were serially diluted, and aliquots (1 mL) were pour-plated in Sabouraud Dextrose Agar, supplemented with chloramphenicol (0.1 g/L) for enumeration of yeasts and molds, M17 agar supplemented with glucose (5 g/L *w*/*v*), for coccus-shaped LAB, and modified de Man, Rogosa, and Sharpe (mMRS) agar, supplemented with maltose (5 g/L), fresh yeast extract (50 mL/L), adjusted to pH 5.6, for rod-shaped LAB. M17 and mMRS agar media were also supplemented with 0.1 g/L cycloheximide after sterilization. Colonies of presumptive yeasts and LAB were counted after incubation at 30 °C for 48 h [28].

In addition, yeast extract agar (yeast extract 3 g/L, tryptone 6 g/L, agar 15 g/L, pH 7.2) was employed to enumerate psychrophilic and mesophilic heterotrophic microorganisms in both tap water and grape water, after incubation at 22 °C for 72 h (psychrophilic) and at 37 °C for 48 h (mesophilic) [28].

The detection limit for plate count was 1 log CFU/mL (or g). Calibration standards for each agar medium are reported in the Supplementary Table S1.

Germinated seeds and bread underwent microbiological analyses, after homogenization and serial dilution, as described for doughs/sourdough samples. Total aerobic mesophilic microorganisms were enumerated on Plate Count Agar, incubated at 30 °C for 48 h. Total coliforms were estimated using Violet Red Bile Glucose agar, incubated at 37 °C for 24 h [20].

### 2.6. Descriptive Sensorial Analysis of Bread

A panel test was conducted at the Department of Plant, Soil, and Food Sciences at the University of Bari Aldo Moro to qualitatively describe sensory traits of bread samples. The panel consisted of 10 assessors (6 females and 4 males) with ages spanning from 27 to 47 years. The panelists underwent two training sessions, which encompassed tasting bread samples that differed from those used in the panel test, and familiarization with sourdough bread enriched with germinated seeds, aimed at establishing a standardized sensory vocabulary. A total of 22 sensory attributes, covering aspects such as appearance (crumb and crust color, crust thickness, crust appearance, crumb structure), odor (yeasty, fermentation, toasted, vegetables, legume-like, nutty, off-odor), texture (elasticity, dryness, hardness, softness, graininess, compactness, crust crunchiness), and taste (bitter, salty, legume-like, acidic, overall taste) were considered [29]. Furthermore, panelists were encouraged to suggest novel descriptive terms to augment the existing attributes. During the assessment, the bread samples were sliced to a 1.5 cm thickness and served at 22 °C under standard lighting conditions. To mitigate any potential bias, samples were coded with unique 3-digit identifiers and presented in a randomized sequence [30]. Each panelist had three different samples of each type of bread and rated the sensory attributes on a non-structured 15 cm line scale using sheets describing the vocabulary for each attribute, with scores ranging from 0 (not detected) to 15 (extremely strong), adhering to ISO 13299:2010 standard [31] for sensory analysis.

### 2.7. Specific Volume, Image, and Color Analysis of Bread Samples

The specific volume of bread samples was measured through the rapeseed displacement method AACC 10-05.01 [32]. In addition, bread crumbs were examined utilizing image analysis technology. Pictures of the sliced bread were scanned (at 300 dpi) through an Image Scanner (Amersham Pharmacia Biotech, Uppsala, Sweden). Full-scale images were gray-scaled, and two sub-images (1001 × 1393 pixels, field of view) were analyzed using the UTHSCSA ImageTool application (version 2.0, University of Texas Health Science Centre, San Antonio, TE, USA). Gas cells and non-cells were distinguished using a threshold approach (threshold value 130) [33]. Gas cell area in the bread crumb was calculated by the software as a percentage ratio between the area filled by gas cells (black pixels) and the total area. A Minolta CR-10 camera was used to assess color in three separate locations on the bread crust and crumb [34]. It was performed using the *L*, a*, and b* color space analysis approach, where *L* stands for lightness (white to black) and a* (red-green) and b* (yellow-blue) for chromaticity coordinates.

### 2.8. Nutritional Analysis of Bread

Nutritional analysis of breads included determination of proteins (total nitrogen x 6.25), lipids, ashes, and water content, according to the AOAC methods 978.04, 2003.06, 930.05, and 930.04, respectively. Total available carbohydrates were calculated as the difference [100 − (proteins + ashes + lipids + water)]. Total dietary fibers were determined using the Total Dietary Fiber (Megazyme kit (Bray, Ireland), following the AACC 32-05.01 method. Energy value was calculated based on the amount of lipids, proteins, and total carbohydrates, multiplied by the corresponding energy value of each nutrient [35].

### 2.9. Statistical Analyses

All experiments were conducted in triplicate, and results were expressed as mean ± standard deviation. Statistical analyses were performed using SPSS Statistics version 28. Data were subjected to one-way analysis of variance (ANOVA) to assess significant differences among samples. When ANOVA indicated significant effects (*p* < 0.05), means were compared using Tukey’s Honestly Significant Difference (HSD) post hoc test. Spearman correlations for color indices and scores attributed to bread crust and crumb were computed using Statistica v. 7.0.

## 3. Results

### 3.1. Microbial Cell Densities Differed Between Tap Water and Grape Water

**Figure 2:**
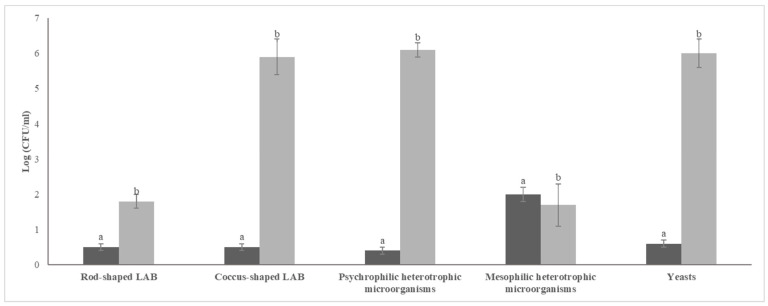
Cell densities (expressed in log CFU/mL) of presumptive LAB, yeasts, psychrophilic and mesophilic heterotrophic microorganisms found in tap water (dark gray bars) and grape water (light gray bars) used in the first sourdough fermentation. For a given microbial group, bars showing different letters are significantly different (*p* < 0.05).

The microbiological analysis revealed that grape water exhibited significantly higher (*p* < 0.05) counts of all the targeted microbial groups, except for heterotrophic mesophilic microorganisms. The latter were found at higher (*p* < 0.05) cell density in tap water (Figure 2). Grape water was dominated (at an order of magnitude of 6 log CFU/mL) by psychrophilic heterotrophic microorganisms, presumptive yeasts, and presumptive coccus-shaped LAB.

### 3.2. Boosted Sourdough Gave Similar Performances to Control Sourdough

**Figure 3:**
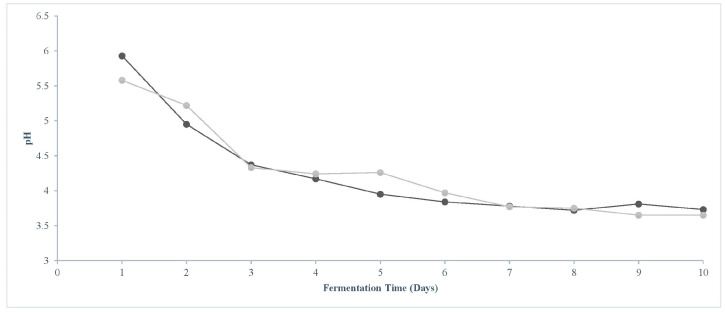
Values of pH of control (dark gray plot) and boosted (light gray plot) sourdoughs after each daily fermentation. Error bars are not visible because error values were below 0.07.

The pH of both CSD and BSD sourdoughs steadily declined throughout the 10-day fermentation period (Figure 3), with both falling below 4.5 on day 3. pH reached about 3.8 on day 7 in both sourdough samples, and then it remained constant. The acidification rate was slightly higher in the CSD (0.24 pH units/day) than in the boosted sourdough (0.22 pH units/day).

**Figure 4:**
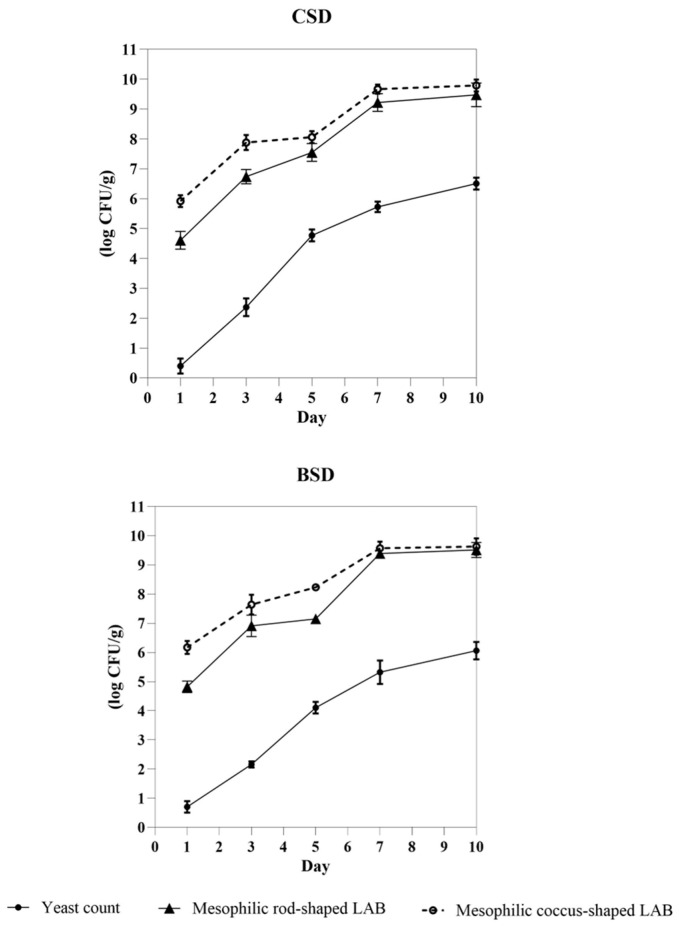
Cell densities (log CFU/g) of presumptive yeasts, rod-shaped LAB, and coccus-shaped LAB in the control sourdough (CSD) and boosted sourdough (BSD) after each daily fermentation.

From the beginning of fermentation until day 3, BSD exhibited significantly (*p* < 0.05) higher counts of presumptive coccus-shaped and rod-shaped LAB than the control (Figure 4). After the first fermentation, presumptive yeasts were detected at very low cell density in both sourdough samples, with no significant difference (*p* > 0.05) between BSD and CSD. At day 3, CSD harbored a higher (*p* < 0.05) cell density of yeast than BSD, although in both sourdough samples, yeasts were in the order of magnitude of 2 log CFU/g. Yeasts increased in both sourdough samples after 7 and 10 days to 5 and 6 log CFU/g, respectively, with CSD characterized by higher (*p* < 0.05) cell density than BSD (Figure 4). Presumptive LAB increased until day 7 up to 9 log CFU/g; afterwards, they did not increase. Both on days 7 and 10, no significant (*p* > 0.05) difference in cell densities of LAB was found between BSD and CSD.

### 3.3. Germinated Lentil and Bread Wheat Seeds Were Selected as Additional Ingredients for Bread

**Figure 5:**
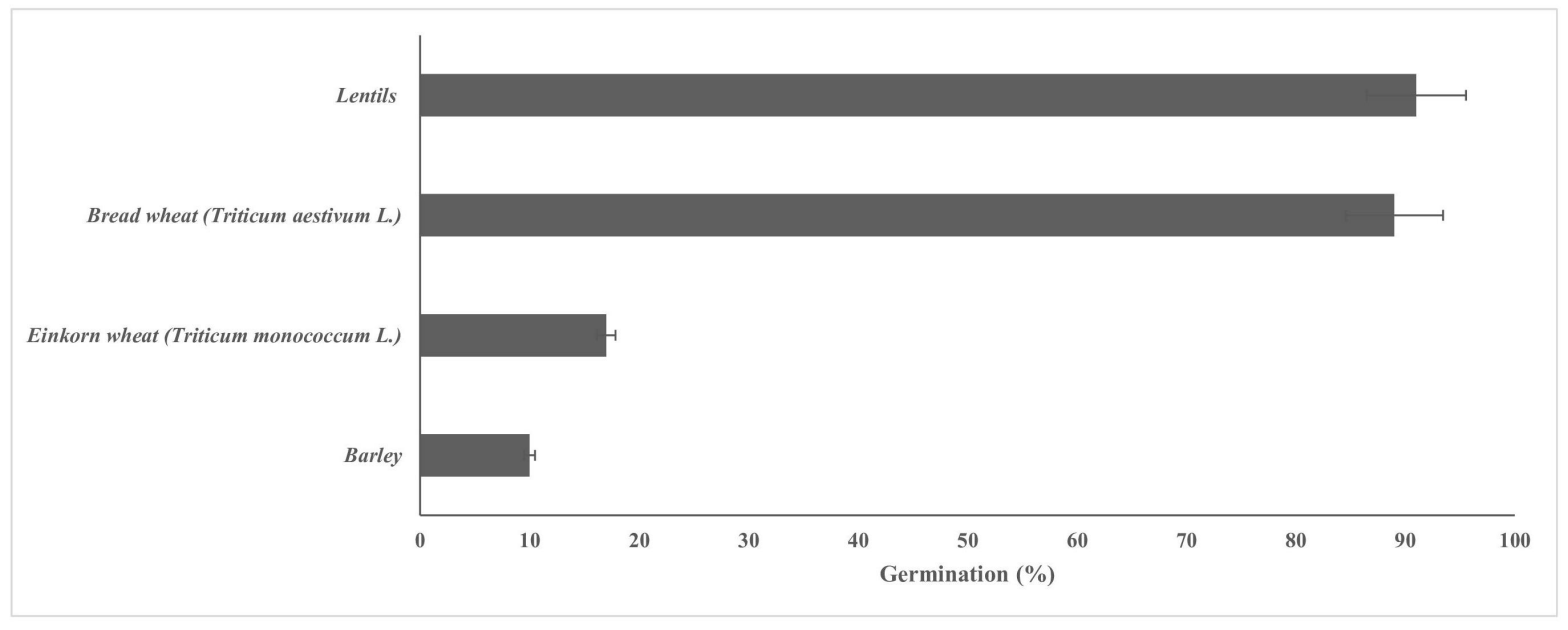
Percentage of germination, determined after 24 h at 25 °C, of lentil, bread wheat, einkorn wheat, and barley seeds.

Germination tests showed that, after 24 h at 25 °C, lentils and bread wheat exhibited the highest germination percentages (Figure 5 and Figure S1). Einkorn wheat and barley, showing germination percentages lower than 20%, were discarded from further experiments. Microbiological analyses showed that germinated lentils and bread wheat harbored total coliforms at an order of magnitude of 8 log CFU/g, with no significant difference between them. Total mesophilic aerobic bacteria were found at higher (*p* < 0.05) cell density in germinated lentils (8.2 log CFU/g) than in germinated bread wheat (7.8 log CFU/g).

### 3.4. Sourdough Bread Was Free from Total Coliforms

Preliminary results of specific volume suggested using, besides sourdough, baker’s yeast as the main leavening agent for bread-making. Three types of bread were manufactured using BSD: (i) with paste from germinated lentils (GL); (ii) with paste from germinated bread wheat (GW); (iii) control. Microbiological analyses performed on bread with paste from either germinated lentils or wheat showed that baking reduced total aerobic mesophilic microorganisms and total coliforms to undetectable levels (<1.0 log CFU/g).

### 3.5. Bread Types Differed in Terms of Specific Sensory Attributes

**Figure 6:**
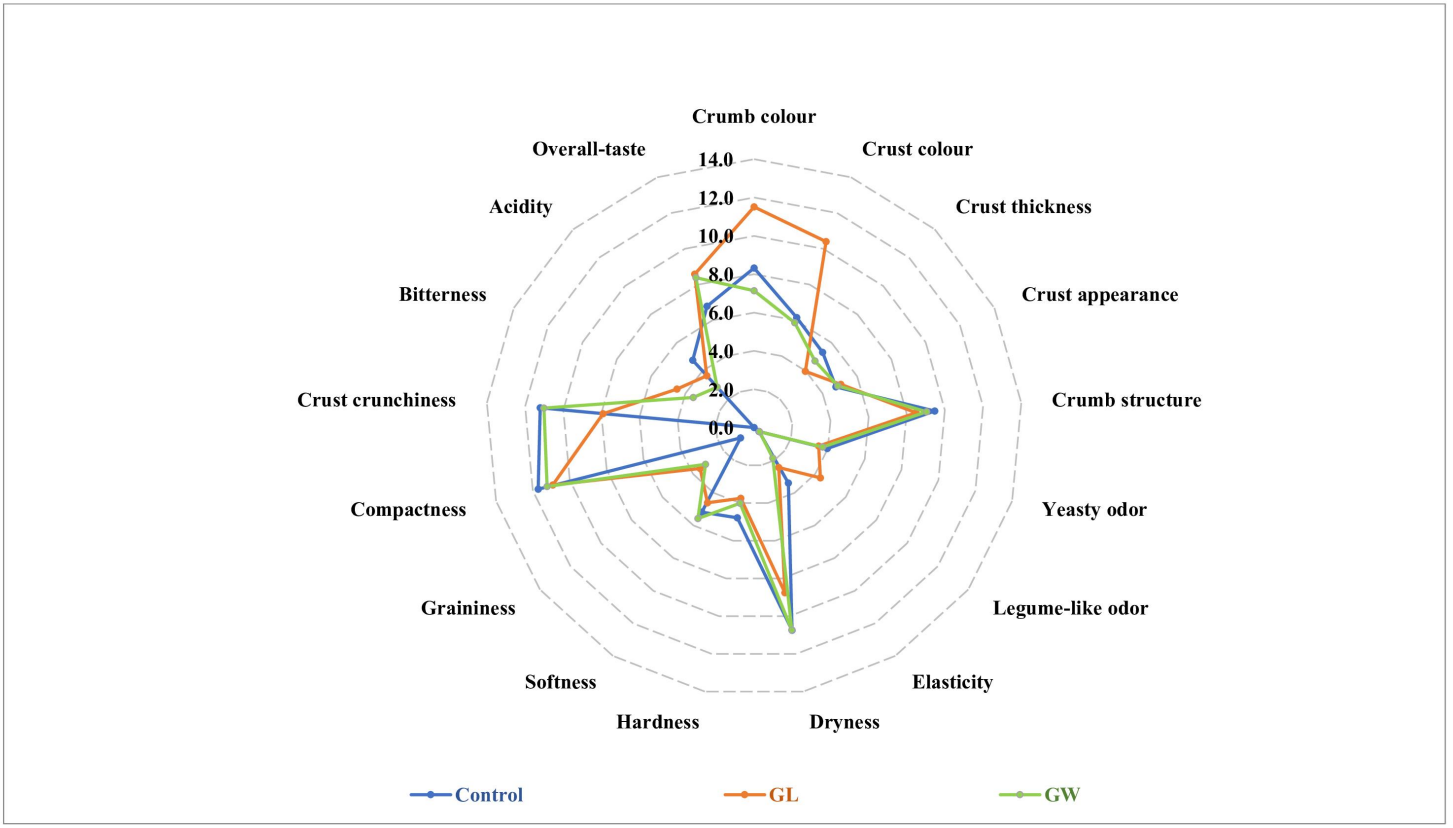
Descriptive sensory analysis of organic sourdough bread, manufactured with germinated lentils (GL) or germinated wheat (GW) paste or without germinated seed paste (Control). Data are the means of scores attributed by ten trained panelists.

The descriptive sensory analysis showed that some attributes received very low scores, with no significant difference between the three types of bread (Supplementary Table S2). For instance, no off-odors were reported in any bread, and this attribute, as well as legume-like taste, was not graphically shown. Control bread showed the highest values for elasticity (3.4 ± 0.5), crust crunchiness (11.2 ± 0.6), and compactness (11.7 ± 0.4). GL received the highest average scores for crumb color (11.5 ± 0.7), crust color (10.4 ± 0.9), and overall taste (8.6 ± 0.8), while also exhibiting higher bitterness (4.5 ± 0.3) and legume-like odor (3.9 ± 0.2) (Figure 6). GW had the highest softness (5.6 ± 0.5) and the lowest bitterness scores (3.5 ± 0.2).

### 3.6. Image Analysis and Color Indexes Differed Among the Bread Types

**Figure 7:**
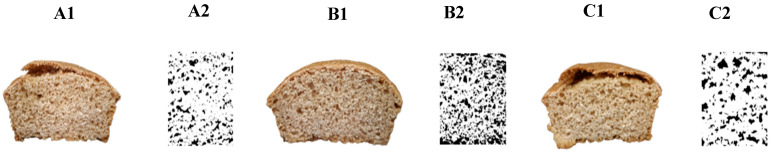
Sections of organic sourdough bread, manufactured without germinated seed paste (control, (**A1**), or with germinated lentils (GL), (**B1**) or germinated wheat (GW), (**C1**) paste. (**A2**,**B2**,**C2**) show crumb subsections, after thresholding to calculate the black pixel area, of control, GL, and GW bread, respectively.

The use of germinated lentils and wheat did not affect the specific volume of sourdough bread (Table 1 and Figure 7). The highest gas cell area was found for GL, whereas control bread was characterized by the lowest values of this crumb parameter. The crumb of GL bread was less light (*p* < 0.05) than control bread, but not significantly different from GW bread. The lowest value for *L* index was found for the crust of GL bread. In addition, this bread showed the highest a* value (Table 1). Correlation analysis between color indexes and scores attributed by the panelists to crumb and crust color showed that the crust color was highly correlated and inversely correlated to a* index and *L* index, respectively (Supplementary Table S3).

### 3.7. Nutritional Analysis of Bread

GL bread was characterized by the lowest and highest values of lipids and proteins, respectively (Table 2). In addition, this bread showed the lowest concentration of carbohydrates. GW showed lipid and protein content that were not significantly different (*p* > 0.05) from the control. GW and control bread showed the highest concentration in total carbohydrates. Total dietary fibers, as well as water content and energy values, were not significantly (*p* > 0.05) different among the three types of bread (Table 2).

## 4. Discussion

In collaboration with a small-medium food enterprise (Perniola Alimenti), we developed a novel organic sourdough bread, enriched with the fresh paste of germinated seeds, aiming to diversify the local bakery food products, in particular bread, that have the potential of targeting consumers interested in products: (i) being entirely manufactured with ingredients from organic farming, including type 1 sourdough as genuine improver; (ii) containing paste from germinated seeds. The experimental plan envisaged the selection of the type of sourdough (produced using either tap water or “grape water”), as well as the selection of seeds among four: lentil, einkorn wheat, barley, and bread wheat. Raisins were chosen to produce grape water as an additional ingredient of sourdough, based on the protocol commonly adopted by the food enterprise. Raisins have been recently used to produce a sourdough starter with scarce leavening capacity [36]. This sourdough was dominated by *Pichia kudriavzevii* (heterotypic synonym: *Candida krusei*) and *Torulaspora delbrueckii*, two yeast species that, compared to *Saccharomyces cerevisiae*, are much less frequently used in bread-making [36]. Under our experimental conditions, we found that grape water was enriched in psychrophilic heterotrophic microorganisms. Values of yeast cell density in grape water suggest that those psychrophilic microorganisms could be represented by yeasts, originating from raisins and transferred into water; in accordance with this finding, grapes putatively transferred a relatively high cell density of yeasts to dough [13]. When analyzing tap water used for producing sourdough, we found that the cell density of mesophilic heterotrophic microorganisms fell in the range (0.8–3.5 log CFU/mL) previously reported [28]. On the contrary, psychrophilic heterotrophic microorganisms in the tap water were lower than the range previously reported [28], although in that study, three out of ten water samples collected in different Italian regions showed counts of psychrophilic heterotrophic microorganisms below the detection limit. While we expected that the use of grape water could have boosted sourdough during its early steps of production, we did not find any difference in yeast cell density between BSD and CSD. On the contrary, BSD was characterized by a higher cell density of LAB in the early production steps, compared to CSD. This result could be explained by hypothesizing that in the sourdough samples subjected to study, yeasts transferred from raisins to water could be less competitive than LAB. It is well known that the competitiveness of microorganisms in sourdough is an essential driver during its preparation [15]. Furthermore, we speculate that the increased LAB cell density in BSD might result from the increased content of fermentable carbohydrates derived from raisins during maceration in water, which might have promoted LAB growth over yeasts. Oshiro et al. [37] also hypothesized that different affinity to different carbohydrates shown by strains of LAB and *S. cerevisiae* could be one of the bases for competition between LAB and yeasts in early steps of sourdough preparation. Under our experimental conditions, a pH less than 4.5 was reached at the end of the third fermentation, regardless of the use of grape water. On the contrary, we had previously reported that the use of water from pear maceration had favored a pH decrease to 4 already after the second fermentation [14]. This could be explained considering that raisins and pears may differ in terms of microbial community and concentration of sugars. Additionally, in the previous research, 100 g of pears were subjected to maceration [14] vs. 50 g of raisins processed in the present study. After 10 fermentation steps, we found that the use of grape water instead of tap water did not affect LAB cell density and their main metabolic activity, resulting in dough acidification. On the contrary, we found slightly (although significantly different) higher yeast cell density in CSD than in BSD, which could be due to the different composition (in terms of species and strains) of the microbial community, driven by the two different water types. Both sourdough types showed values of pH, cell densities of LAB and yeasts, falling in the typical range for traditional sourdough [15]. Further experiments focused on the use of BSD for bread-making because this kind of sourdough had been produced according to the protocol commonly used by the food enterprise that participated in this study. In addition, use of grape water for preparing sourdough could be helpful in all cases wherein flour does not harbor pro-technological microorganisms [38]; in such cases, indeed, a longer time would be needed to obtain mature sourdough.

In parallel with comparison between BSD and CSD, we considered four seeds that, after germination, could be used as a paste and as additional ingredients of organic sourdough bread. We selected lentils and bread wheat, based on their higher percentage of germination, compared to barley and einkorn. Germination percentage could be affected by various factors, such as time and conditions of storage, eventual treatments of seeds, such as decortication, as well as parameters (e.g., temperature) applied during germination protocol [39]. Use of germinated seeds in food is consolidated in many African and Asian populations because they improve the sensory properties [40] and texture of food items [41], in addition to enhancing the nutritional value [41,42]. Wheat bread based on 20% replacement with flour from germinated oat was characterized by increased bioactive compounds (gamma aminobutyric acid, total polyphenols, free phenolic acids) and decreased starch digestibility [43]. Bread entirely based on sprouted wheat flour showed higher *in vitro* protein digestibility than bread made of regular flour [44]. Chemical modifications occurring during seed germination typically cause an increase in microbial load [45,46]. Therefore, in the current study, germinated lentils and bread wheat grains were subjected to microbiological analyses, which showed high cell density of presumptive *Enterobacteriaceae* in accordance with previous studies [46,47,48]. However, the use of fresh germinated lentils/wheat, as a paste, as additional ingredients of organic sourdough bread did not raise any safety concerns. Indeed, after baking, *Enterobacteriaceae* were not detected, possibly because of the high temperature reached by bread during baking, in accordance with previous studies [49,50]. Seeds subjected to germination for being used as ready-to-eat food are usually contaminated, especially by *Salmonella* spp. and Shiga-toxin producing *Escherichia coli*, which may survive for long periods during seed storage [27]. In this study, we demonstrated that bread-making is a viable option for consuming germinated seeds, ensuring the food is free from microbiological hazards.

The three sourdough bread types (GL, GW, and control) considered in this study were subjected to a panel test, since the food enterprise collaborating with researchers was interested in understanding the potential of bread with germinated seeds in view of scaling up the process. Indeed, previous studies reported that the use of flour from germinated seeds was able to modify the sensory attributes of bread. Atudorei et al. [51] found that adding 5–7.5% germinated lentil flour improved bread porosity, crumb color, and sensory appeal. Badawy et al. [52] reported that 6–9% germinated wheat flour enhanced aroma and crumb color. In the current study, we found that, compared to the control, sourdough bread incorporating 5% of germinated lentils/wheat grains received a higher score for overall taste and softness, respectively. In addition, panelists perceived the crumb and especially the crust of sourdough GL bread as more colored than the two other bread types. Higher scores of graininess and overall taste attributed to GL and GW breads could be explained by the use of 5% germinated seeds for two main reasons: (i) proteins, lipids, and minerals contained in the additional ingredient; (ii) the germination process itself leads to increased concentrations of enzymes and chemical compounds affecting sensory/texture properties [51]. The color analysis showed that GL bread had the lowest value of *L* index (lightness) and the highest value of a* index (meaning color of crust approaching red). Overall, considering bread crust, the score attributed by the panelists to color was strongly correlated with a* index, but highly and inversely correlated with *L* index.

Use of germinated lentils/wheat grains did not affect the specific volume of bread. Yet, image analysis showed that sourdough bread with paste from germinated wheat and, mainly, lentils, was characterized by a higher percentage of gas cell area than the control. These results are in agreement with a previous study that reported that the addition of 5% flour from 48 to 72 h germinated bread wheat did not affect the specific volume. However, the resulting bread appeared with a different size and distribution of gas cells, with respect to the control bread [53].

From a nutritional perspective, control bread and GW could be labeled as “rich in fiber” (at least 6% of fiber), whereas GL could be labeled as a “source of fiber” (at least 3% of fiber) [54]. However, the use of germinated lentils as additional ingredients in sourdough bread resulted in providing more proteins and less lipids than the two other bread types. These findings are consistent with those reported by Hernández-Aguilar et al. [55], who developed wheat breads with 5% and 10% germinated lentil flour. They observed that incorporating 5% lentil flour increased the protein content to approximately 11.8% compared to 10.5% in the control, while the lipid content decreased from 12.16% to 10.3%. Another study also found that the addition of 5–10% roasted-sprouted lentil flour to gluten-free bread (corn/rice based) resulted in 77–90% higher protein than the control bread [56].

## 5. Conclusions

This study showed that the addition of paste from germinated lentils into organic sourdough bread enhanced nutritional quality and overall taste, while maintaining desirable technological characteristics. On-going research aims to evaluate the feasibility of scaling up the protocol (sourdough production, seed germination and blending, bread-making) to small–medium enterprises and consumers’ acceptance of the novel bread. Further studies may be conceived aiming to (i) adapt the protocol to other underutilized seeds; (ii) assess the most appropriate storage conditions for paste from germinated seeds; and (iii) evaluate the impact of using germinated seeds paste on the shelf-life of bread.

## Figures and Tables

**Table 1:** Specific volume, gas cell areas, and values of color indexes of organic sourdough bread, manufactured with germinated lentils (GL), or germinated wheat (GW) paste, or without germinated seed paste (control).

Bread	Specific Volume (cm^3^/g)	Gas Cells Area (%)	Crust Color			Crumb Color		
			*L*	a*	b*	*L*	a*	b*
Control	2.2 ± 0.2 ^a^	14.1 ± 0.5 ^c^	59.6 ± 1.0 ^a^	9 ± 0.5 ^b^	23.7 ± 0.8 ^a^	62.3 ± 0.1 ^a^	4.4 ± 0.2 ^a^	16.9 ± 0.2 ^a^
GL	2.2 ± 0.1 ^a^	28.6 ± 0.8 ^a^	52.1 ± 0.4 ^b^	11.0 ± 0.6 ^a^	24.2 ± 0.5 ^a^	56.4 ± 1.4 ^b^	4.5 ± 0.1 ^a^	17 ± 0.4 ^a^
GW	2.2 ± 0.1 ^a^	18.1 ± 0.8 ^b^	58.4 ± 0.9 ^a^	8.5 ± 0.5 ^b^	22.5 ± 0.9 ^a^	58.9 ± 2.1 ^ab^	4.5 ± 0.4 ^a^	18 ± 1 ^a^

^a–c^ Values in the same column with different superscript letters are significantly different (*p* < 0.05).

**Table 2:** Nutritional analysis of organic sourdough bread, manufactured without germinated seeds (control), with germinated lentils (GL), or germinated wheat (GW) paste.

Bread	Lipids (%)	Proteins (%)	Water (%)	Total Carbohydrates (%)	Dietary Fibers (%)	Energy Value (kcal/100 g)
Control	0.4 ± 0.1 ^a^	8.6 ± 0.1 ^b^	37.9 ± 0.1 ^a^	44.8 ± 0.1 ^a^	6.6 ± 1.2 ^a^	243 ± 1.0 ^a^
GL	0.3 ± 0.1 ^b^	9.9 ± 0.1 ^a^	37.4 ± 0.2 ^a^	43.3 ± 0.1 ^b^	5.7 ± 0.5 ^a^	245 ± 1.0 ^a^
GW	0.4 ± 0.1 ^a^	8.8 ± 0.1 ^b^	37.0 ± 0.1 ^b^	45.5 ± 0.1 ^a^	6.2 ± 0.6 ^a^	247 ± 1.0 ^a^

^a,b^ Values in the same column with different superscript letters are significantly different (*p* < 0.05).

## Data Availability

The original contributions presented in the study are included in the article/Supplementary Materials, further inquiries can be directed to the corresponding author.

## Supplementary Materials

The following supporting information can be downloaded at: https://www.mdpi.com/article/10.3390/foods14183263/s1, Figure S1: Lentil (A), bread wheat (B), einkorn wheat (C), and barley (D) seeds after germination (24 h at 25 °C). Supplementary Table S1. Calibration standards of microbiological analyses used in the current study. Supplementary Table S2. Sensory analysis of organic sourdough bread, manufactured with germinated lentils (GL) or germinated wheat (GW) paste or without germinated seed paste (Control). Data values are the means of scores attributed by ten trained panelists. Values in the same column with different superscript letters (a,b) are significantly different (*p* < 0.05). Supplementary Table S3. Correlation coefficients between values of color indexes (*L*, *a**, *b**) of crust and crumb and the sensory panel evaluation scores of organic sourdough bread (GL, GW and control).

## Abbreviations

The following abbreviations are used in this manuscript:

GL	Bread made with addition of germinated lentils paste
GW	Bread made with addition of germinated wheat paste
LAB	Lactic acid bacteria
CSD	Control sourdough
BSD	Boosted sourdough
G%	Germination percentage
CFU	Colony forming unit
