# Supplementary material for: Development of Organic Sourdough Bread with Paste from Germinated Seeds
Source: Foods. 2025 Sep 20;14(18):3263. doi: 10.3390/foods14183263 (PMC12469423; doi:10.3390/foods14183263)
Supplement: Supplementary file 1 [file foods-14-03263-s001.zip › foods-3808065-supplementary.pdf]

Figure S1. Lentil (A), bread wheat (B), einkorn wheat (C), and barley (D) seeds after germination (24 h at 25 °C).

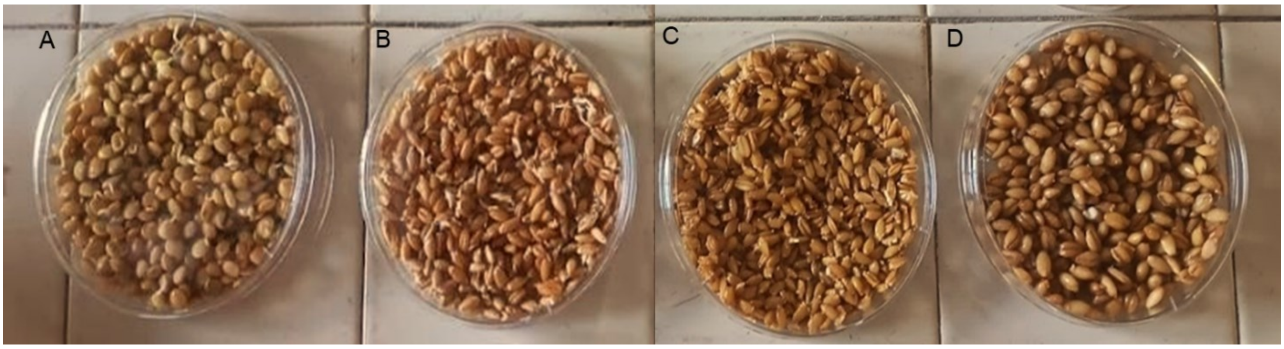

Supplementary Table S1. Calibration standards of microbiological analyses used in the current study

Agar medium	Calibration standard
**Sabouraud Dextrose**	*Candida albicans* ATCC 10231
**M17**	*Streptococcus thermophilus* ATCC 14485
**Modified MRS**	*Lactobacillus gasseri* ATCC 19992
**Yeast Extract**	*Escherichia coli* ATCC 25922
**Plate Count**	Not available
**Violet Red Bile Glucose**	*Escherichia coli* ATCC 25922

Supplementary Table S2. Sensory analysis of organic sourdough bread, manufactured with germinated lentils (GL) or germinated wheat (GW) paste or without germinated seed paste (Control). Data values are the means of scores attributed by ten trained panelists. Values in the same column with different superscript letters (a–b) are significantly different ($P < 0.05$).

	Descriptors	Control	GL	GW
**Visual**	Crumb color	$8.3{\pm}0.9^{b}$	$11.5{\pm}0.3^{a}$	$7.1{\pm}0.4^{b}$
	Crust color	$6.2{\pm}0.4^{b}$	$10.4{\pm}0.3^{a}$	$5.9{\pm}0.6^{b}$
	Crust thickness	$5.3{\pm}0.4^{a}$	$4.0{\pm}0.5^{b}$	$4.7{\pm}0.5^{a}$
	Crust appearance	$4.8{\pm}0.6^{a}$	$5.1{\pm}0.2^{a}$	$4.9{\pm}0.3^{a}$
	Crumb structure	$9.5{\pm}0.5^{a}$	$8.5{\pm}0.6^{a}$	$9.0{\pm}0.9^{a}$
**Olfactory**	Yeasty	$4.0{\pm}0.5^{a}$	$3.5{\pm}0.5^{a}$	$3.7{\pm}0.3^{a}$
	Legume-like	$0.3{\pm}0.2^{b}$	$4.3{\pm}0.2^{a}$	$0.4{\pm}0.3^{b}$
**Structure**	Elasticity	$3.4{\pm}0.5^{a}$	$2.4{\pm}0.3^{b}$	$1.9{\pm}0.3^{b}$
	Dryness	$10.8{\pm}0.4^{a}$	$8.8{\pm}0.5^{b}$	$10.7{\pm}0.9^{a}$
	Hardness	$4.8{\pm}0.7^{a}$	$3.7{\pm}0.4^{a}$	$4.0{\pm}0.4^{a}$
	Softness	$5.2{\pm}0.5^{a}$	$4.6{\pm}0.3^{a}$	$5.6{\pm}0.5^{a}$
	Graininess	$0.9{\pm}0.5^{b}$	$3.5{\pm}0.4^{a}$	$3.2{\pm}0.8^{a}$
	Compactness	$11.7{\pm}0.4^{a}$	$10.9{\pm}0.5^{a}$	$11.2{\pm}0.5^{a}$
	Crust crunchiness	$11.2{\pm}0.6^{a}$	$7.9{\pm}0.5^{b}$	$11.0{\pm}0.5^{a}$
**Taste**	Bitterness	0.0^{b}	$4.5{\pm}0.1^{a}$	$3.5{\pm}0.9^{a}$
	Acidity	$4.8{\pm}0.8^{a}$	$3.7{\pm}0.5^{a}$	$2.9{\pm}0.4^{b}$
	Overall-taste	$6.8{\pm}0.8^{b}$	$8.6{\pm}0.3^{a}$	$8.4{\pm}0.3^{a}$

Supplementary Table S3. Correlation coefficients between values of color indexes (*L*, *a**, *b**) of crust and crumb and the sensory panel evaluation scores of organic sourdough bread (GL, GW and control).

	**r**
L crust vs. Score attributed to crust color	-0.978
*a** crust vs. Score attributed to crust color	0.992
*b** crust vs. Score attributed to crust color	0.767
L crumb vs. Score attributed to crumb color	-0.638
*a** crumb vs. Score attributed to crumb color	0.254
*b** crumb vs. Score attributed to crumb color	-0.651
